# Reversible Switching of Single-Molecule Magnetic Behaviour by Desorption/Adsorption of Solvent Ligand in a New Dy(III)-Based Metal Organic Framework

**DOI:** 10.3389/fchem.2021.714851

**Published:** 2021-08-05

**Authors:** Xiao-Jiao Song, Zhao-Bo Hu, Miao-Miao Li, Xin Feng, Ming Kong, Xiao-Ming Xue, Yi-Quan Zhang, You Song

**Affiliations:** ^1^ State Key Laboratory of Coordination Chemistry, School of Chemistry and Chemical Engineering, Nanjing University, Nanjing, China; ^2^ Key Laboratory of National Forestry and Grassland Administration on Wildlife Evidence Technology, School of Criminal Science and Technology, Nanjing Forest Police College, Nanjing, China; ^3^ Chaotic Matter Science Research Center, Department of Materials, Metallurgy and Chemistry, Jiangxi University of Science and Technology, Ganzhou, China; ^4^ Jiangsu Key Lab for NSLSCS, School of Physical Science and Technology, Nanjing Normal University, Nanjing, China

**Keywords:** single-molecule magnet, Dy(III)-based, metal organic framework, induced by coordination solvent, reversible on/off switch

## Abstract

Two metal-organic frameworks (MOFs), [Dy(BDC)(NO_3_)(DMF)_2_]_*n*_ (**1**, H_2_BDC = terephthalic acid) and [Dy(BDC)(NO_3_)]_*n*_ (**1a**), were synthesized. The structures of MOFs **1** and **1a** are easy to be reversibly transformed into each other by the desorption or adsorption of coordination solvent molecules. Accordingly, their magnetic properties can also be changed reversibly, which realizes our goals of manipulating on/off single-molecule magnet behaviour. MOF **1** behaves as a single-molecule magnet either with or without DC field. Contrarily, no slow magnetic relaxation was observed in **1a** both under zero field and applied field.

## Introduction

Molecule-based magnetic materials are increasingly favoured by researchers for their potential applications in information storage, quantum computers and spintronics (Sessoli et al., 1993; Wernsdorfer and Sessoli, 1999; Wang et al., 2010; Kirk et al., 2013; Woodruff et al., 2013; Pei et al., 2018; Yu et al., 2019). Among them, the magnetic switch based on single-molecule magnets (SMMs) or single-chain magnets (SCMs) is one of the hot topics in this field (Hoshino et al., 2012; Fetoh et al., 2016; Dickie et al., 2017; Shao et al., 2018; Cador et al., 2019; Ma et al., 2020). Constructing SMMs or SCMs whose structure can change reversibly is an effective way to obtain magnetic switches. As is well known, most SMMs and SCMs are low-dimensional coordination compounds (Ishikawa et al., 2003; Woodruff et al., 2013), and their structures often produce an irreversible collapse when manipulating the magnetic properties by tuning molecular structures. Until now, there are only a few reports on magnetic switches based on SMMs or SCMs induced by reversible structural transformation (Suzuki et al., 2013; Zhang et al., 2015; Wu et al., 2017; Xin et al., 2019; Hojorat et al., 2020; Zhu et al., 2020; Hu et al., 2021).

Contrary to the low-dimensional SMM or SCM systems, metal-organic frameworks (MOFs) usually exhibit higher structural stability, which is more conducive to the realization of reversible structural change (Yaghi et al., 2003; Kitagawa et al., 2004; Guo et al., 2006; Aulakh et al., 2015). This advantage allows them to be efficient platforms for developing magnetic switching materials. However, it is difficult for most 3D-MOFs to show slow magnetic relaxation because there are frequently existing exchange interactions and magnetic order (Miyasaka et al., 2006; Bernot et al., 2009). For lanthanide MOFs, the exchange interaction between lanthanide ions is generally weak due to the effective shielding of unpaired electrons in the 4f orbital of the lanthanide ions (Aulakh et al., 2015; Liu et al., 2015; Liu et al., 2016; Iwami et al., 2017; Castells-Gil et al., 2018). Furthermore, 4f ions contain various coordination geometries, most from six-to nine-coordination, which contribute to the design and adjustment of the structures (Ruiz-Martínez et al., 2008; Song et al., 2012; Aulakh et al., 2015; Guo et al., 2017; Li et al., 2017; Wang et al., 2018). In addition, the quantum tunneling effect can be effectively suppressed by the weak couplings, especially weak ferromagnetic couplings, thereby improving energy barriers, in lanthanide MOFs (Woodruff et al., 2013; Das et al., 2018; Ji et al., 2019). Therefore, lanthanide ions are well suited for constructing MOFs with SMM behaviour, in particular for those with magnetic switching effects. For example, Li and co-workers reported the switching of SMM behaviour in Dy-MOF system by changing the coordination geometry of the Dy(III) ions (Zhou et al., 2013). It should be noted that most of these MOFs show antiferromagnetic coupling between the adjacent 4f ions (Baldovi et al., 2014; Yi et al., 2015; Huang et al., 2018; Zhang et al., 2018). On account of the above, the design and synthesis of 4f ion-based MOFs which exhibit weak ferromagnetic coupling between metal centers is a good choice for obtaining magnetic switches.

Herein we report a Ln-MOF, [Dy(BDC)(NO_3_)(DMF)_2_]_n_ (**1**), obtained from the reaction of terephthalic acid (H_2_BDC) with Dy(NO_3_)_3_·6H_2_O, which shows slow relaxation behaviour. Since there are no free solvent molecules in this complex, it is a good platform to study the effect of changes in coordination geometry on slow relaxation behaviour. Interestingly, the magnetic interaction between the 4f metal centers shows a transition from ferromagnetic coupling to antiferromagnetic coupling and the slow magnetic relaxation phenomenon also disappears with the loss of coordination DMF solvent molecules in this complex.

## Experimental

### Synthesis of [Dy(BDC)(NO_3_)(DMF)_2_]_n_ **(1)**

A mixture of H_2_BDC (23 mg, 0.137 mmol) and Dy(NO_3_)_3_·6H_2_O (62.56 mg, 0.137 mmol) in 1.25 ml EtOH/DMF (*V*:*V* = 1:4) solution was sealed in a 15 ml Schlenk glass tube. To remove air, the Schlenk tube with reaction solution was purged and backfilled with argon gas three times, then heated in an oven at 100°C for 36 h. After the temperature was gradually reduced to room temperature, the colourless bulk crystals were obtained, and the yield was about 36% calculated based on Dy(III) ion. Anal. calcd. for C_14_H_18_DyN_3_O_9_: C, 31.44%; H, 3.39%; N,7.86%. Found: C, 31.52%; H, 3.45%; N, 7.79%.

### Synthesis of [Dy(BDC)(NO_3_)]_n_ **(1a)**

The collected crystals of complex **1** were washed with ethanol, and dried in air. After then, the crystals were heated in an oven at 170°C for 24 h, complex **1a** was obtained. Anal. calcd. for C_8_H_4_DyNO_7_: C, 24.73%; H, 1.04%; N,3.60%. Found: C, 24.70%; H, 1.10%; N, 3.68%.

### Synthesis of [Dy_0.1215_Y_0.8785_(BDC)(NO_3_)(DMF)_2_]_n_ **(1@Y)**

The colourless bulk crystals of complex **1@Y** were obtained following the procedure described for complex **1** except that Dy(NO_3_)_3_·6H_2_O was replaced by Dy(NO_3_)_3_·6H_2_O and Y(NO_3_)_3_·6H_2_O in a 1:10 M ratio. The accurate ratio of Dy/Y is 1:7.23 in the magnetically diluted complex **1@Y**, which was determined by X-ray fluorescence spectrometry (Supplementary Figure S1). Elemental Anal. Calcd. for C_14_H_18_Dy_0.1215_Y_0.8785_N_3_O_9_: C, 35.76%; H, 3.86%; N, 8.94%. Found: C, 35.30%; H,3.89%; N, 8.63%.

### X-Ray Data Collection and Structure Refinement

The diffraction data for **1** were collected on a Bruker Smart CCD area-detector diffractometer using Mo-*K*α radiation (*λ* = 0.71073 Å) in the *ω*-scan mode at 296 K. The diffraction data were treated using SAINT, and absorption corrections were applied by using SADABS. All the non-hydrogen atoms were located by Patterson’s method using the SHELXS program of the SHELXTL package and by subsequent Fourier syntheses (Sheldrick, 2008). The hydrogen atoms were determined theoretically and treated using a riding model. The hydrogen atoms were refined with isotropic thermal parameters. All non-hydrogen atoms were refined by full-matrix least-squares on *F* ^2^ with anisotropic thermal parameters. All the calculations were performed by the SHELXTL-2014 program (Sheldrick, 2015). The details for the structural analyses of complex **1** are shown in Supplementary Table S1. The selected bond distances and angles for complex **1** are listed in Supplementary Table S2. The CCDC number of complex **1** is 2059079.

## Results and Discussion

### Synthesis and Characterization

Infrared (IR) spectra were recorded as KBr pellets under vacuum condition. Complex **1a** was immersed in DMF solvent for 3 days, it changed to complex **1-back**. To study the stability of the framework and the loss of coordinated DMF molecules, the data at variable temperatures were collected for complex **1** and **1-back**. The temperature-dependent IR spectra of complex **1** and **1-back** were shown in Supplementary Figures S2, S3, respectively. The bands at 1,623 and 1,038 cm^−1^ are assigned to the C=O stretching vibration (*ν*_CO_) and the CH_3_ rocking region (*r*_CH3_) of DMF molecules, respectively (Freire and Alves, 2015; Ohashi and Takeshita, 2021). The peaks of 1,623 and 1,038 cm^−1^ are disappeared when the temperature reached 175°C, which is due to the losing of DMF molecules. Except for some slight differences, such as a decomposed component of the *ν*_CO_ band at 1,686 cm^−1^ (extremely small), the IR spectra of complex **1** and **1-back** are almost the same (Supplementary Figure S4). The slight differences in IR spectra may be due to the perturbation of high temperature in the coordination environment. In addition, the temperature-dependent IR spectrum of complex **1-back** is also consistent with that of complex **1.** The results of IR spectra support that complex **1a** can uptake DMF molecules and transform back to complex **1**. The thermogravimetric analyses were performed in N_2_ atmosphere at a heating rate of 10°C min^−1^ from 30°C to 800°C for complex **1** and **1-back** (Supplementary Figure S5). There is no weight loss before 140°C for complex **1** and **1-back**. For complex **1**, it reveals a weight loss of 27.31% between 140°C and 295°C, which corresponds to the loss of two coordination DMF molecules (27.33%). Then it shows a continued weight loss in the temperature range of 295–800°C, which is due to the collapse of the framework. For complex **1-back**, there is a weight loss of 26.87% between 140°C and 295°C, which is slightly lower than the loss of two coordination DMF molecules (27.33%). A continued weight loss in the temperature range of 295–800°C is also due to the collapse of the framework. The results of thermogravimetric analysis for complex **1-back** are consistent with those for complex **1**. The recorded experimental PXRD pattern of **1** and **1@Y** agree well with the simulated pattern from single-crystal X-ray diffraction data of **1,** which confirms the phase purity for the microcrystal of **1** and **1@Y** (Supplementary Figure S6). There are some slight differences between the experimental PXRD pattern of complex **1a** and that of complex **1,** which means that the framework of complex **1a** is slightly deformed after high-temperature treatment.

### Crystal Structure

**FIGURE 1:**
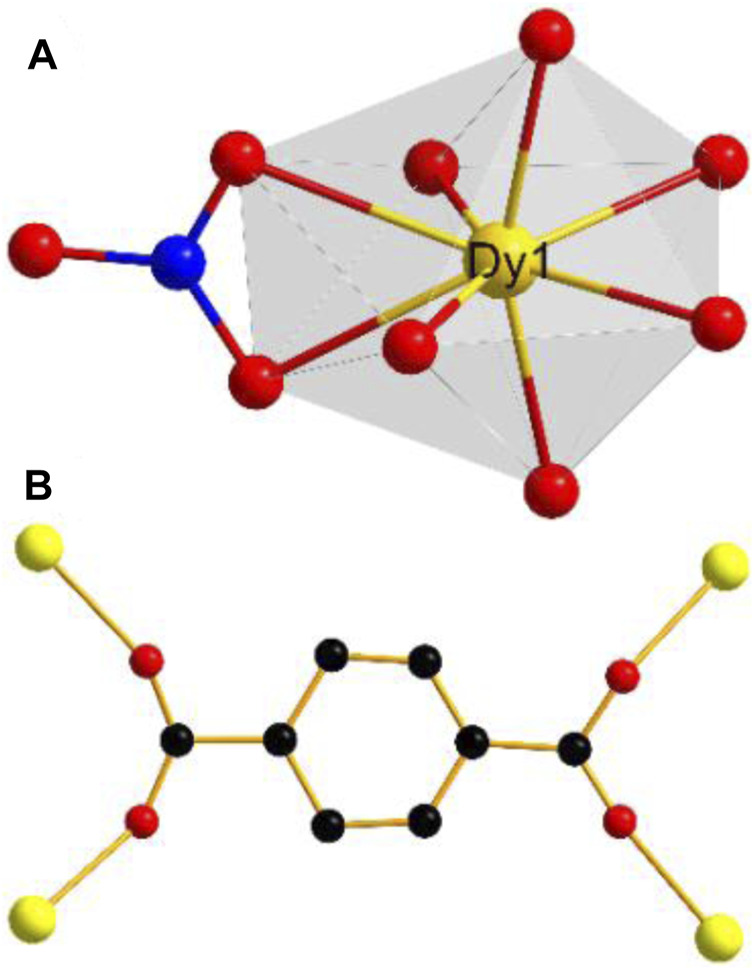
**(A)** Coordination geometries of Dy(III) ion; **(B)** Coordiantion modes of BDC^2-^ ligands of **1**. Color code: C, black; N, blue; O, red; Dy, yellow.

**FIGURE 2:**
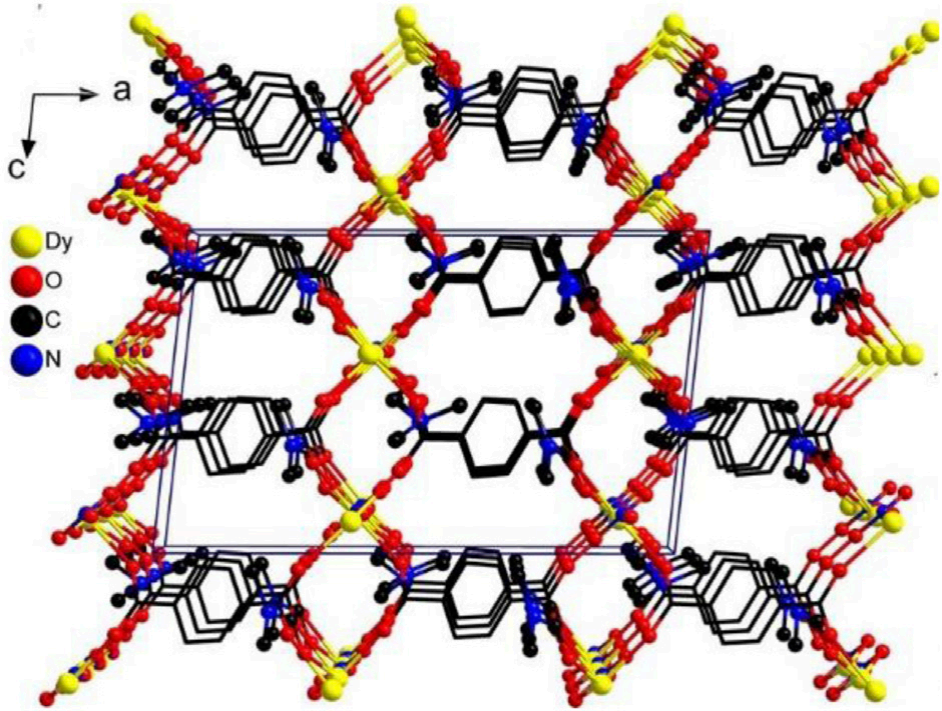
The framework of complex **1.**

The result of X-ray single-crystal diffraction indicates that complex **1** belongs to the monoclinic space group *C*2/*c*. There are one Dy(III) ion, one BDC^2−^ ligand, one nitrate ion and two DMF molecules in the asymmetric unit of **1**. The Dy(III) ion is eight-coordinated, in which four BDC^2−^ ligands provide four oxygen atoms, one nitrate provides two oxygen atoms, and two DMF molecules provide two oxygen atoms for coordination. The Dy(III) center adopts a snub disphenoid (JSD-8) coordination geometry (Figure 1A), which was analyzed by the SHAPE 2.1 software, and the calculated results are listed in Supplementary Tables S3, S4 (Llunell et al., 2013). All bond lengths and bond angles are within the normal range. Each ligand BDC^2−^ catches four metal Dy(III) ions (Figure 1B). In this way, the adjacent Dy(III) ions are linked together, forming a one-dimensional chain along the *c* axis (Supplementary Figure S7). These chains are further bridged by the ligand BDC^2−^, giving rise to a three-dimensional network structure. Interestingly, there are no free solvent molecules in the three-dimensional channel because the coordinated DMF solvent molecules are filled into the pores (Figure 2). In order to realize the function of the magnetic switch, we studied the influence of the presence or absence of DMF molecules in the framework. After heating 24 h in an oven at 170°C, the coordinated DMF solvent molecules were removed. Not only the DMF molecules are absent in the pores, but also the number of coordination atoms around the Dy(III) center has changed. This means that the magnetic properties of complex **1a** are different from those of complex **1**. Complex **1a** has been putted into DMF solvent for the purpose of proving the structural reversibility. Complex **1-back** was obtained by putting complex **1a** into DMF solvent for 3 days. As expected, the recorded experimental PXRD patterns of **1-back** is consistent with the simulated pattern of complex **1**. These results further indicate that complex **1** can transform to complex **1a** by heating and then comeback to complex **1** by putting complex **1a** into DMF solvent.

### Magnetic Properties

**FIGURE 3:**
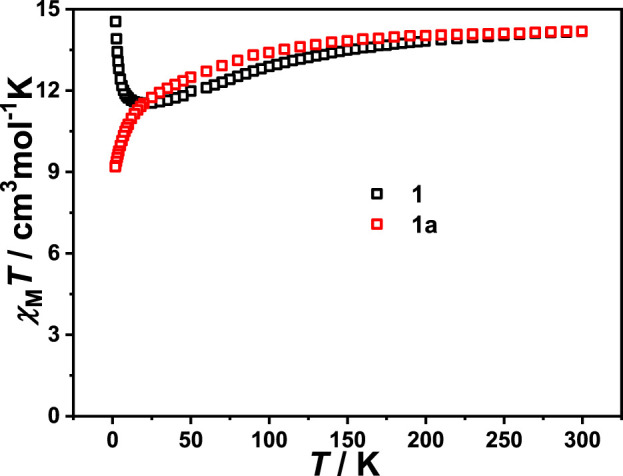
The plots of *χ*_M_ *T* versus *T* for **1** and **1a** under an applied field of 1 kOe.

Variable temperature susceptibility measurements were carried out in a temperature range of 1.8–300 K under a DC field of 1.0 kOe. The plot of *χ*_M_ *T* versus *T* for a [Dy(BDC)(NO_3_)(DMF)_2_] unit is shown in Figure 3. The product *χ*_M_ *T* of complex **1** is 14.18 cm^3^ K mol^−1^ at room temperature, which is in agreement with the theoretical value of 14.167 cm^3^ K mol^−1^ for single Dy(III) ions (*S* = 5/2, *L* = 5, *J* = 15/2, *g* = 4/3). Upon cooling, the *χ*_M_ *T* value of complex **1** gradually decreases and reaches a minimum of 11.51 cm^3^ K mol^−1^ at 25 K. This phenomenon could be ascribed to the depopulation of Stark sublevels of Dy(III) ion. As the temperature continues to decrease, the *χ*_M_ *T* value increases rapidly and reaches a maximum of 14.54 cm^3^ K mol^−1^ at 1.8 K, which indicates the presence of ferromagnetic coupling between Dy(III) ions (Aulakh et al., 2015). For complex **1a**, the value of *χ*_M_ *T* is 14.19 cm^3^ K mol^−1^ at 300 K, which is closed to the theoretical value for one Dy(III) ion. Upon cooling, the *χ*_M_ *T* value decreases slowly in the high-temperature region, then decreases rapidly and reaches 9.19 cm^3^ K mol^−1^ at 1.8 K, which is owing to the depopulation of Stark sublevels and/or the antiferromagnetic coupling between adjacent Dy(III) ions.

The field-dependence magnetizations of **1** and **1a** were measured in the whole field (0–7 T) at the temperature from 1.8 to 10 K (Supplementary Figures S8, S9). For complex **1**, the magnetization *M* reaches a saturated value (5.73 *μ*_B_) at 7 T, which is larger than the observed value (5.23 *μ*_B_) for one anisotropic Dy(III) ion (Tang et al., 2006). This phenomenon indicates that there is also a strong magnetic anisotropy in complex **1**. Besides, the non-superposition of the *M* vs. *H*/*T* curves provides further evidence for the presence of strong magnetic anisotropy in this system (the inset of Supplementary Figure S8). For complex **1a**, the value of *M* is still not saturated at 7 T.

### AC Magnetic Measurements

**FIGURE 4:**
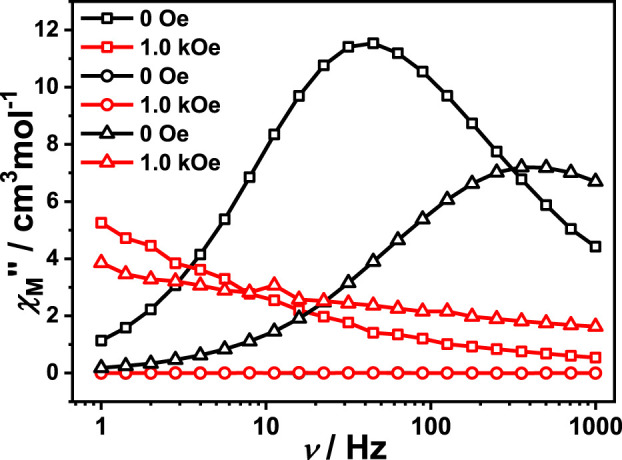
Both 0 Oe and 1.0 kOe field measurement performed on polycrystalline sample of complex **1** (□), **1**a (○) and **1-back** (∆), respectively.

**FIGURE 5:**
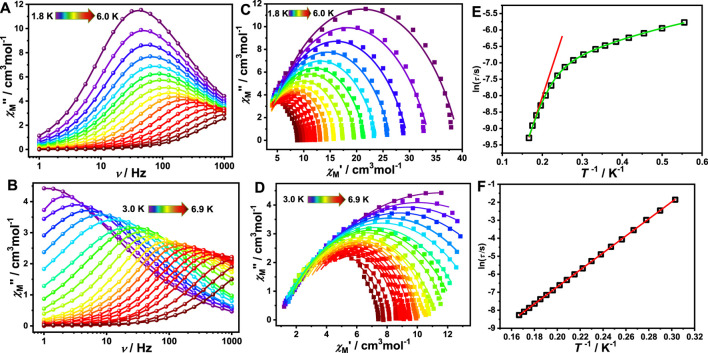
Frequency-dependent of the out-of-phase (χ″) under zero DC-field **(A)** and under 1,000 Oe **(B)** of complex **1**; Cole−Cole curves under zero DC-field **(C)** and under 1,000 Oe **(D)** of complex **1**. Solid lines represent the best fit with Debye model. Plot of ln (*τ*/s) versus *T* ^−1^ under zero DC-field **(E)** and under 1,000 Oe **(F)** for complex **1**, where the red solid line represents the fitted results using the Arrhenius formula and the green solid line represents the fitted results using Eq. 1.

The alternating current (AC) magnetic susceptibility of complexes **1**, **1a** and **1-back** were measured to investigate their dynamic magnetic behaviour. For complex **1**, the AC magnetic susceptibility measurements were done under a zero DC field and 1 kOe DC field. Obvious out-of-phase signals were observed in both cases, indicating that complex **1** exhibits SMM behaviour (Supplementary Figure S10). However, no out-of-phase signal was observed both under zero field and 1 kOe DC field, indicating that complex **1a** does not show SMM behaviour (Supplementary Figure S11). Interestingly, complex **1-back** exhibits an obvious out-of-phase signal (Supplementary Figure S12). The literatures demonstrate that subtle modification of solvent, auxiliary ligand, coordination environment and inter-molecular interaction have a significant impact on the magnetic dynamics of lanthanide single-molecule magnets (Zhang et al., 2016; Zhang et al., 2019; Kong et al., 2020). Compared with complex **1**, the coordination environment may be slightly different in complex **1-back** which has undergone the process of removing and absorbing DMF molecules, resulting in a difference of the magnetic relaxation (Figure 4). For complex **1**, SMM behaviour disappears by removing the coordinated DMF molecules, and appears when recovering DMF molecules. In short, reversible switching of SMM behaviour is realized by desorption/adsorption of coordinated DMF molecules. In the whole tested DC field, only one relaxation process was observed for complex **1**. In order to study the slow relaxation behaviour, both zero field and 1.0 kOe were chosen to test the dynamic magnetization due to the longest relaxation time (Supplementary Figures S13, S14). In the given fields and temperature ranges, the variable-frequency *χ*_M_″ is shown in Figure 5 for **1**. The Cole-Cole plots are fitted through the Debye model using CCFIT software (Guo et al., 2011). The extracted *α* values are listed in Supplementary Tables S5, S6. In zero DC field, the effective energy barrier is 37.01 (3) K with *τ*_0_ = 1.98 × 10^–7^ s by fitting with the Arrhenius formula. The ln (*τ*) vs. *T* ^−1^ curve indicates possible multiple slow relaxation processes, which is described in Eq. 1. The best resulting parameters are *τ*_QTM_ = 0 s, *C* = 143.95 K^−1.59^ s^−1^, *n* = 1.59, *τ*_0_ = 9.82 × 10^–8^ s and *U*_eff_ = 43.02 K. However, in 1 kOe DC field, the ln(*τ*) vs. *T* ^−1^ curve is replaced by a straight line, which indicates that it only has the Orbach process. The effective energy barrier *U*_eff_ is equal to 47.27 K with *τ*_0_ = 9.62 × 10^–8^ s. It can be seen from Supplementary Table S7 that the quantum tunnelling effect (QTM) cannot be suppressed by antiferromagnetic coupling between neighbouring Dy(III) ions. However, ferromagnetic coupling between neighbouring Dy(III) ions may effectively suppress QTM. In this work, the QTM is also not observed, which proves the conclusion that the ferromagnetic interaction can suppress QTM. In order to further prove this conclusion, the alternating current (AC) magnetic susceptibility of diamagnetically diluted sample **1@Y** was measurement under zero DC field (Supplementary Figure S15). In the low-temperature region, the peak values of the *χ*_M_″ vs. *ν* curves does not move with increasing temperature, which indicates that there is an obvious QTM process in complex **1@Y**. The Cole-Cole plots of **1@Y** are fitted by the Debye model using CCFIT software (Supplementary Figure S16, Supplementary Table S8). The ln(*τ*) *vs*. *T* ^−1^ curve indicates possible multiple slow relaxation processes, so the data are fitted using the Eq. 1 which includes QTM, Orbach and Raman processes (Supplementary Figure S17). The best resulting parameters are *τ*_QTM_ = 420.37 s, *C* = 3.32 K^−5.71^ s^−1^, *n* = 5.71, *τ*_0_ = 1.37 × 10^–8^ s and *U*_eff_ = 41.00 K. The fitting result proves that there is a QTM process in complex **1@Y**. These results further prove that the ferromagnetic interaction leads to the disappearance of the quantum tunneling process in complex **1**.

$$\tau^{-1}=\tau_{QTM}^{-1}+CT^{n}+\tau_{0}^{-1}\exp\left(\frac{-U_{eff}}{k_{B}T}\right)$$ (1)

ab initio calculations

$$J_{dip}=-\frac{\mu_{B}^{2}g_{1Z}g_{2Z}}{r^{3}}\left(\cos\theta -3\cos\varphi_{1}\cos\varphi_{2}\right)$$ (2)

To gain further insights into the magnetic coupling between neighbouring Dy(III) ions for complex **1**, CASSCF calculations based on X-ray single-crystal structure were performed using MOLCAS 8.4 program (Aquilante et al., 2016) and SINGLE_ANISO programs (Chibotaru et al., 2008a; Chibotaru et al., 2008b; Ungur et al., 2009). A Dy(III) ion was randomly selected from complex **1**, and the principal magnetic axe of this ground Dy(III) ion was calculated (Supplementary Figure S18). The calculated energy levels (cm^−1^) and *g* (*g*_*x*_, *g*_*y*_, *g*_*z*_) tensors of the minimum KDs of the Dy (III) motif for complex **1** are shown in Supplementary Table S9. The calculated values of the correlative tensors in the ground state (*m*_*J*_ = ±15/2) are 0.002 (*g*_*x*_), 0.002 (*g*_*y*_) and 19.893 (*g*_*z*_), respectively. The results show a strong axial anisotropy in the ground state for complex **1**, which leads to a slow magnetic relaxation behaviour in a zero field for complex **1**. For complex **1**, the *m*_J_ values of the ground states are mostly composed of ±15/2, and the predominant *m*_*J*_ values of the first excited states are ±13/2 (Supplementary Table S10). The calculated energy of the first excited states is 192.4 cm^−1^. The value of the experimental energy barrier (47.27 K) is much smaller than the calculated value, suggesting that such a relaxation does not reach the first excited state due to fast under-barrier relaxation which is induced by anharmonic phonons (Lunghi et al., 2017; Kong et al., 2020) (Supplementary Figure S19; Supplementary Table S10). The principal magnetic axes of Dy(III) ions are parallel to each other based on the structure and symmetry of complex **1** (Supplementary Figure S20). According to Eq. 2, the calculated value of *J*_dip_ is 0.48 cm^−1^. The calculation details are presented in the Supplementary Material. The small *J*_dip_ value proves that the magnetic interaction between neighboring Dy(III) ions is too weak to influence the intrinsic magnetic properties of complex **1**.

## Conclusion

MOF **1** was obtained based on Dy(III) ions, H_2_BCD and DMF, which shows slow magnetic relaxation behaviour. Removing the coordinated DMF molecules from MOF **1** by heating, MOF **1a** can be obtained. MOF **1a** can be back to MOF **1** by being immersed into DMF solvent, which has been proved by FT-IR, TGA, SXRD, PXRD and magnetic property. We have proved that MOFs based on Dy(III) ions achieved reversible on/off switching of SMM behaviour induced by coordination DMF solvent molecules. This phenomenon demonstrates that MOFs could be powerful platforms for studying both the structural transformation and magnetic properties.

## Data Availability

The datasets presented in this study can be found in online repositories. The names of the repository/repositories and accession number(s) can be found in the article/Supplementary Material.
