# Supplementary material for: Reversible Switching of Single-Molecule Magnetic Behaviour by Desorption/Adsorption of Solvent Ligand in a New Dy(III)-Based Metal Organic Framework
Source: Front Chem. 2021 Aug 5;9:714851. doi: 10.3389/fchem.2021.714851 (PMC8374150; doi:10.3389/fchem.2021.714851)
Supplement: Supplementary file 1 [file DataSheet2.pdf]

Supplementary Material

Materials and General Methods.

All chemicals were used as commercially obtained without further purification. Elemental analysis (C, H, and N) was performed with a Heraeus CHN-Rapid elemental analyzer. The ratio of Dy/Y was determined with XRF (EDAX Orbis). FT-IR spectra were recorded with a Bruker Tensor 27 spectrometer using the reflectance technique (1800-600 cm^{-1}) under vacuum condition. Samples were prepared using KBr disks. TGA were performed using a Perkin-Elmer thermal analyzer from 30 to 800 ℃ under dry N_2 at a heating rate of 10 ℃ min^{-1}. Powder X-ray diffraction (XRD) intensities were measured on a Rigaku D/max diffractometer (Cu-Kα = 1.54056 Å). All magnetic measurements were carried out on a Quantun Design MPMS SQUID-VSM. Complete-active-space self-consistent-field (CASSCF) calculations were performed using the MOLCAS 8.4 program package.

Table S1 Crystallographic data and structural refinement parameters for complex **1**

	**1**
Formula	$C_{14}H_{18}DyN_3O_9$
f w	534.81
T / K	296(2)
λ / Å	0.71073
Crystal system	Monoclinic
Space group	$C2/c$
a / Å	17.13(4)
b / Å	10.80(3)
c / Å	10.69(4)
α / °	90
β / °	95.80(4)
γ / °	90
V / Å^3	1968(10)
Z	4
D_c / g cm^{-3}	1.805
μ / mm^{-1}	3.847
F(000)	1044
θ / °	2.233 to 25.009
Reflns collected, Reflns unique	5135,1722
R_{int}	0.0409
GOF on F^2	1.063
R_1 [I> 2σ(I)][a], R_1 (all data)[a]	0.0279, 0.0289
wR_2 [I>2σ(I))][b], wR_2 (all data)[b]	0.0683, 0.0692
Largest diff. Peak, hole / (e Å^{-3})	0.632 , -0.952

[a]$R_1 = \Sigma ||Fo| - |Fc||/\Sigma|Fo|$; [b]$wR_2 = [\Sigma w(Fo^2 - Fc^2)^2/\Sigma w(Fo^2)^2]^{½}$

Table S2 Bond lengths [Å] and angles [deg] for complex **1**

Bond lengths and Angles for complex **1**			
O3-Dy1A	2.272(6)	Dy1-O4B	2.431(6)
Dy1-O5	2.332(6)	Dy1-O2B	2.525(7)
Dy1-O5B	2.332(6)	Dy1-O2	2.525(7)
Dy1-O4	2.431(6)	Dy1-N1	2.940(11)
C4-O3-Dy1A	163.7(3)	O4-Dy1-O4B	71.0(3)
O3C-Dy1-O3A	159.2(3)	O3C-Dy1-O2B	75.6(2)
O3C-Dy1-O5	96.2(3)	O3A-Dy1-O2B	125.19(18)
O3A-Dy1-O5	90.2(3)	O5-Dy1-O2B	73.5(2)
O3C-Dy1-O5B	90.2(3)	O5B-Dy1-O2B	73.90(18)
O3A-Dy1-O5B	96.2(3)	O4-Dy1-O2B	134.5(2)
O5-Dy1-O5B	144.0(2)	O4B-Dy1-O2B	140.9(2)
O3C-Dy1-O4	78.5(2)	O3C-Dy1-O2	125.18(18)
O3A-Dy1-O4	84.6(2)	O3A-Dy1-O2	75.6(2)
O5-Dy1-O4	72.9(2)	O5-Dy1-O2	73.90(18)
O5B-Dy1-O4	142.92(14)	O5B-Dy1-O2	73.5(2)
O3C-Dy1-O4B	84.6(2)	O4-Dy1-O2	140.9(2)
O3A-Dy1-O4B	78.5(2)	O4B-Dy1-O2	134.5(2)
O5-Dy1-O4B	142.92(14)	O2B-Dy1-O2	49.7(3)
O5B-Dy1-O4B	72.9(2)		

Symmetry codes: A) -*x* + 1, -*y*, -*z*; B) -*x* + 1, *y*, -*z* + 1/2; C) -*x* + 1/2, -*y* - 1/2, -*z*; D) *x*, -*y*, *z* + 1/2.

Table S3 The possible geometries of octa-coordination metal centers

Geometry	Point group	Polyhedron
OP-8	D_{8h}	Octagon
HPY-8	C_{7v}	Heptagonal pyramid
HBPY-8	D_{6h}	Hexagonal bipyramid
CU-8	O_h	Cube
SAPR-8	D_{4d}	Square antiprism
TDD-8	D_{2d}	Triangular dodecahedron
JGBF-8	D_{2d}	Johnson - Gyrobifastigium (J26)
JETBPY-8	D_{3h}	Johnson - Elongated triangular bipyramid (J14)
JBTP-8	C_{2v}	Johnson - Biaugmented trigonal prism (J50)
BTPR-8	C_{2v}	Biaugmented trigonal prism
JSD-8	D_{2d}	Snub disphenoid (J84)
TT-8	T_d	Triakis tetrahedron
ETBPY-8	D_{3h}	Elongated trigonal bipyramid (see 8)

Table S4 Deviation parameters calculated by SHAPE from each ideal polyhedron for complex **1**. The best matches are displayed in blue

Geometry	Dy1
OP-8	15.625
HPY-8	17.923
HBPY-8	14.917
CU-8	17.15
SAPR-8	5.473
TDD-8	6.35
JGBF-8	12.438
JETBPY-8	24.715
JBTP-8	5.046
BTPR-8	5.255
**JSD-8**	**4.412**
TT-8	17.82
ETBPY-8	21.515

Table S5 The fit parameters obtained from analyses of the ac susceptibilities of **1** under zero field

T / K	χ_T / cm^3 mol^{-1}	χ_S / cm^3 mol^{-1}	ln(τ / s)	α	R^2
1.8	39.3835	2.79055	-5.77119	0.27954	0.2734118
2	33.8557	2.66307	-5.94837	0.27547	0.2555323
2.2	29.7654	2.55035	-6.10076	0.2709	0.1439272
2.4	26.5424	2.43529	-6.23761	0.26793	0.1346719
2.6	23.9519	2.35683	-6.35765	0.26537	0.1275883
2.8	21.7858	2.30561	-6.47484	0.26032	0.1235353
3	19.9824	2.27459	-6.58597	0.25411	0.203956
3.3	17.7684	2.27583	-6.7481	0.24039	0.177173
3.6	15.9786	2.29882	-6.92428	0.2205	0.133384
3.9	14.4914	2.34052	-7.13203	0.19147	0.101782
4.2	13.253	2.32449	-7.39161	0.16286	0.165751
4.5	12.2147	2.29749	-7.69094	0.13913	0.14263
4.8	11.3394	2.34302	-7.99378	0.12035	0.29804
5.1	10.5747	2.41484	-8.2985	0.10717	0.19451

5.4	9.90745	2.46517	-8.59958	0.10013	0.13354
5.7	9.32256	2.39068	-8.9117	0.09934	0.09137
6.0	8.80921	2.01111	-9.28663	0.10816	0.06924

Table S6 The fit parameters obtained from analyses of the ac susceptibilities of **1** under 1.0 kOe field

T / K	χ_T / cm^3 mol^{-1}	χ_S / cm^3 mol^{-1}	ln(τ / s)	α	R^2
3.3	22.0432	0.70922	-1.85654	0.49862	0.15847
3.45	19.3208	0.78566	-2.46129	0.46958	0.23828
3.6	17.9775	0.81497	-2.98563	0.45169	0.17849
3.75	16.9016	0.77799	-3.54826	0.4472	0.16727
3.9	15.4325	0.88533	-4.06084	0.42059	0.03225
4.05	14.5281	0.80025	-4.47225	0.41655	0.03907
4.2	13.4379	0.89787	-4.8955	0.39428	0.032061
4.35	12.7784	1.01056	-5.26934	0.37717	0.025657
4.5	12.1459	1.09977	-5.67239	0.35757	0.073337
4.65	11.4469	1.1477	-6.00548	0.35032	0.056134
4.8	11.055	1.43922	-6.3191	0.31368	0.08401
4.95	10.6262	1.61465	-6.60798	0.29432	0.044879
5.1	10.1709	1.94727	-6.89237	0.25367	0.050011
5.25	10.0312	2.03541	-7.15394	0.2598	0.0381
5.4	9.62098	2.34623	-7.38581	0.22198	0.03646
5.55	9.30919	2.4702	-7.63553	0.20653	0.023663
5.7	9.11219	2.5049	-7.87461	0.19739	0.013707

Table S7 Magnetic Interaction and QTM in SMMs based on Dy-MOFs

complex	ferromagnetic	QTM	SMM	ref
$\{[Dy_2(INO)_4(NO_3)_2]\cdot 2DMF\}_n$	**Y**	**Y**	**Y**	1
$\{[Dy_2(INO)_4(NO_3)_2]\cdot 2CH_3CN\}_n$	**Y**	**Y**	**Y**	1
Dy(BTC)	N	Y	Y	2
$Dy(_2O_4)_{1.5}(H_2O)_3]_n\cdot 2nH_2O$	N	Y	Y	3
$\{[Dy(C_2O_4)_{1.5}phen]\cdot 0.5H_2O\}_n$	**Y**	**Y**	**Y**	4
$\{[Dy(OBA)(HOBA)(H_2O)_2]\cdot 3DMF\}_n$	N	Y	Y	5

$[Dy(bipyNO)_4](TfO)_3 \cdot x solvent$	N	Y	Y	6
$\{(H_3O)[Dy(NA)_2] \cdot H_2O\}_n$	N	Y	Y	7
Dy-MBBs	**Y**	**N**	**Y**	8
$[Dy(3\text{-}py\text{-}4\text{-}mc)(C_2O_4)_{0.5}(OH)(H_2O)]$	**Y**	**N**	**Y**	9
$\{[Dy_2(MSA)_3(H_2O)_4] \cdot 3H_2O\}_n$	Y	N	Y	10
$[Dy(MMA)(INA)(H_2O)_2]_n$	N		N	11
$\{[Dy((NH_2)_2\text{-}bdc)_{1.5}(DMF)_2] \cdot DMF \cdot H_2O\}$	N	Y	Y	12
$\{[Dy(ant)((NH_2)_2\text{-}bdc)_{0.5}(DMF)_2] \cdot DMF \cdot H_2O\}_n$	N	Y	Y	12
$\{[Dy_2(cyim)_3(DMF)_4] \cdot DMF\}_n$	N	Y	Y	12
$\{(EMIM)[Dy_3(BDC)_5]\}_n$	N	Y	Y	13
$\{[Dy_2(L)_2(\mu_3\text{-}OH)_2(H_2O)] \cdot H_2O\}_n$	**Y**	**N**	**Y**	14
$[Dy(bptc)(phen)(H_2O)]_n$	N	Y	Y	15
$Dy_2(TA)_6(bipy)_2$	**Y**	**N**	**Y**	16
$[Dy_2(TDA)_3(bipy)_2(H_2O)_2] \cdot bipy \cdot 2H_2O$	**Y**	**N**	**Y**	16
$[Dy(H_2O)_2(Habtc)] \cdot 1.7H_2O$	N	Y	Y	17
$[Dy^{III}_4(\mu_3\text{-}OH)_2(\mu_3\text{-}O)_2(cpt)_6(MeOH)_6(H_2O)]_2$	N	Y	Y	18
$[Dy(phen)(L)]_n$	**Y**	**N**	**Y**	19
$[Dy(hfac)_3]_2(PyNO)_3]$	N	Y	Y	20
$[Dy_7(DPA)_5(NA)_3(\mu_3\text{-}OH)_8(H_2O)_3] \cdot 2.5H_2O$	N	Y	Y	21
$\{[Dy(dipp)(dippH)(CH_3OH)(H_2O)_2](CH3OH)_2\}_n$	N	Y	Y	22
$[Dy(\mu\text{-}H_2O)(phen)(\mu\text{-}OH)(nb)_2]_n$	**Y**	**N**	**Y**	23
$Dy_2(L)_2(NO_3)_4(CH_3OH)_2] \cdot 2CH_3OH$	N	Y	Y	24
$[Dy_2(L_1)_2(H_2O)_4(ox)]_n \cdot 4nH_2O$	**Y**	**Y**	**Y**	25
$[Dy_2(L_1)_2(ox)]_n$	N	Y	Y	25
$[Dy(BTB)H_2O]_n$	N	Y	Y	26
$[Dy_2(apca)_4(\mu_2\text{-}OH)_2(H_2O)_2]_n$	**Y**	**N**	**Y**	27
$[Dy(L)Cl(CH_3OH)]_n$	**Y**	**N**	**Y**	28
$[Dy(L)Cl(CH3OH)]_n$	N	Y	Y	29
$\{[Dy(NNO)(glu)] \cdot 0.25H_2O\}_\infty$	N	Y	Y	30
$[Dy_2L_2(H_2O)_5]_n$	N	Y	Y	31
$\{[Dy_2(HCAM)_3(H_2O)_4] \cdot 2H_2O\}_n$	N	Y	Y	32
$[Dy(BDC)(NO_3)(DMF)_2]_n$	**Y**	**N**	**Y**	This work

Table S8 The fit parameters obtained from analyses of the ac susceptibilities of **1@Y** under 1.0 kOe field.

T / *K*	χ_T/cm^3 mol^{-1}	χ_S /cm^3 mol^{-1}	ln(τ / s)	*a*	R^2
1.8	0.60308	3.81026E-15	-6.23948	0.57984	1.85889E-4
2	0.54652	3.57987E-15	-6.39233	0.58449	1.80658E-4
2.2	0.4999	4.72736E-15	-6.59149	0.58278	1.48912E-4
2.4	0.46173	6.76507E-15	-6.81803	0.58093	2.16802E-4
2.6	0.42875	9.37121E-15	-7.09252	0.57524	2.1648E-4
2.8	0.39993	1.01231E-14	-7.40305	0.56837	2.30606E-4
3	0.37607	1.69711E-14	-7.72704	0.56602	2.61961E-4
3.3	0.34551	3.37184E-14	-8.23764	0.56341	2.33189E-4
3.6	0.31969	5.91234E-14	-8.75382	0.56255	1.86377E-4
3.9	0.29719	9.90854E-14	-9.23634	0.55681	1.4327E-4
4.2	0.2776	1.57436E-13	-9.71525	0.55137	1.35186E-4
4.5	0.25947	1.88016E-13	-10.19107	0.54397	7.30053E-5

Table S9 The calculated energy levels (cm^{-1}) and g (g_x, g_y, g_z) tensors of the minimum KDs of Dy(III) motif in complex **1**

KDs	*E* / cm^{-1}	*g*	m_J
1	0.0	0.002<br>0.002<br>19.893	±15/2
2	192.4	0.212<br>0.381<br>17.108	±13/2
3	268.2	0.774<br>1.372<br>17.066	±1/2
4	369.8	1.123<br>3.320<br>11.370	±11/2
5	487.0	1.696<br>3.136<br>8.610	±5/2

6	571.1	8.364<br>7.694<br>4.590	±9/2
7	660.8	0.734<br>1.777<br>16.066	±7/2
8	686.1	0.595<br>0.952<br>19.456	±3/2

Table S10 Wave functions with definite projection of the total moment |m_J> for the lowest two KDs for complex **1**

E / cm^{-1}	wave functions
0.0	99.7%|±15/2>
192.4	85.3%|±13/2>+6.4%|±11/2>

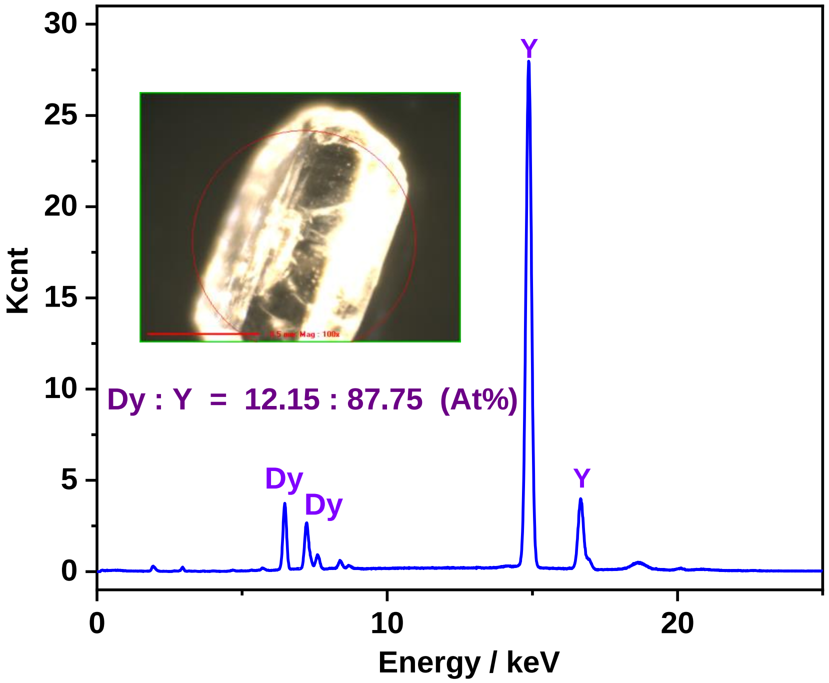

Figure S1 Energy spectra of magnetically diluted complex **1@Y**.

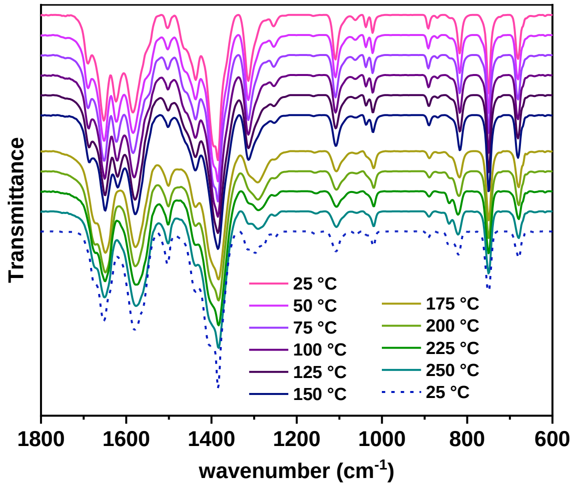

Figure S2 The variable temperature infrared spectra of complex **1**. The blue dashed line represents the infrared spectra of complex **1a** when the temperature cooled down to 25 ℃.

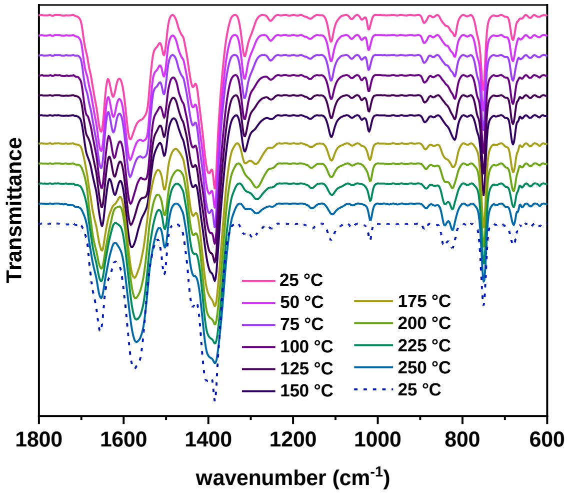

Figure S3 The variable temperature infrared spectra of complex **1-back**. The blue dashed line represents the infrared spectra of complex **1a** when the temperature cooled down to 25 ℃.

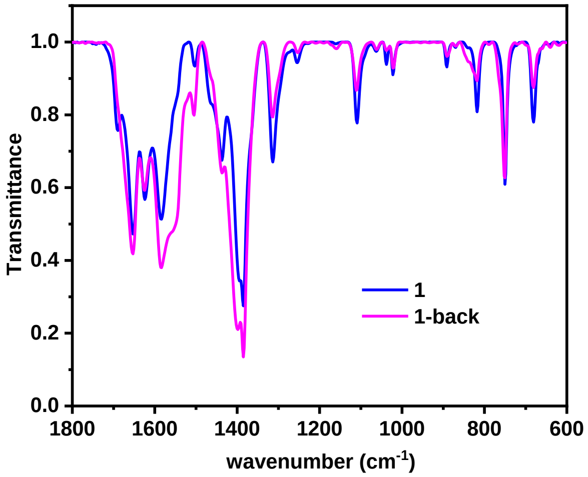

Figure S4 The infrared spectra of complex **1** and **1-back**.

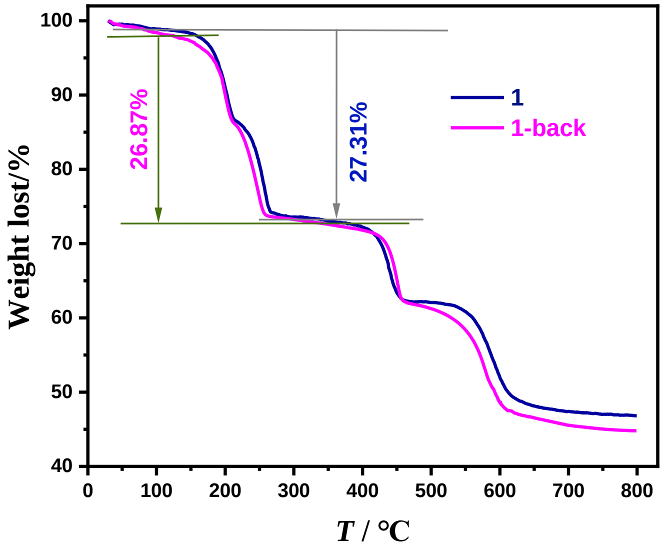

Figure S5 TG curves of **1** and **1-back**.

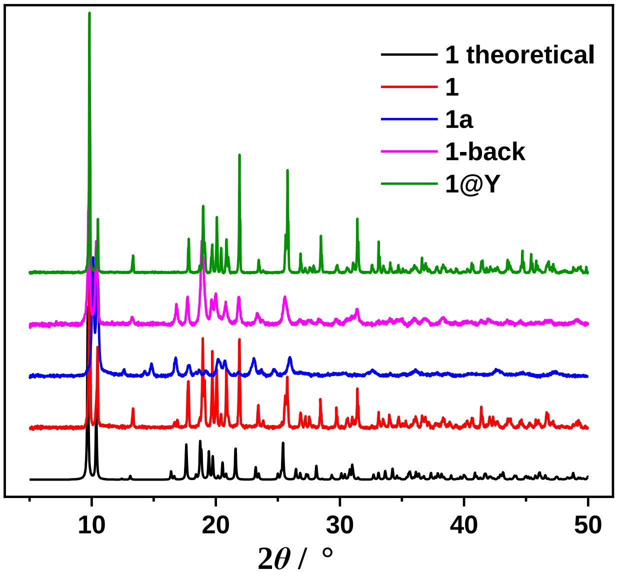

Figure S6 PXRD patterns of **1** theoretical, **1**, **1a**, **1-back** and **1@Y**.

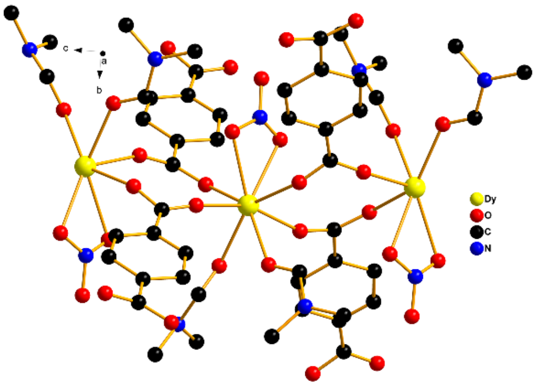

Figure S7 The 1D chain structure of complexes **1**.

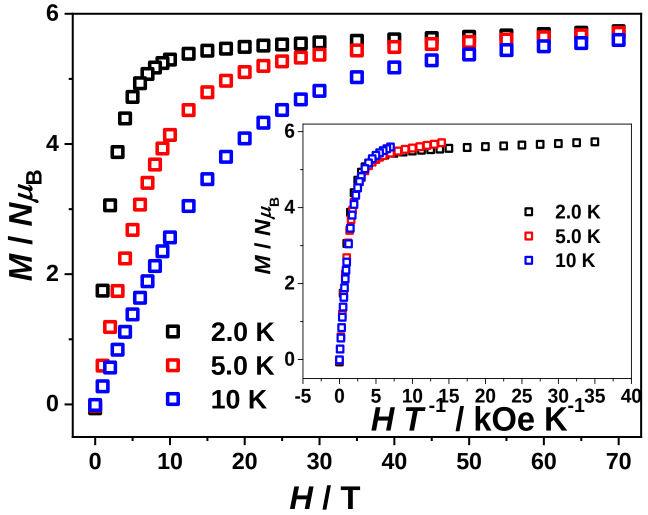

Figure S8 Isothermal magnetization at different temperatures for **1**. Inside: Experimental *M vs. H* / *T* plots at different temperatures for **1**.

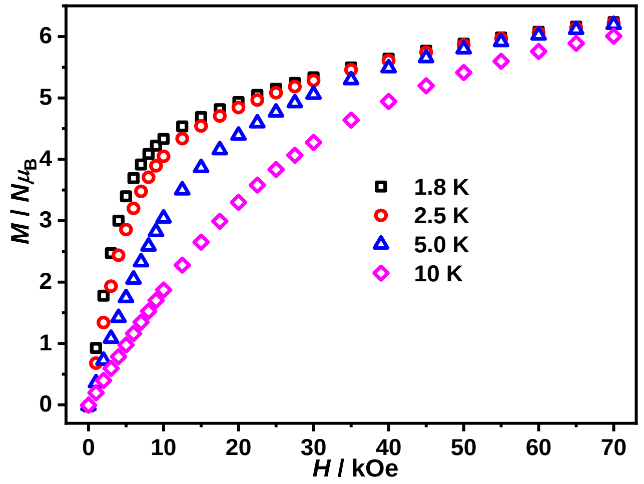

Figure S9 Isothermal magnetization at different temperatures for **1a**.

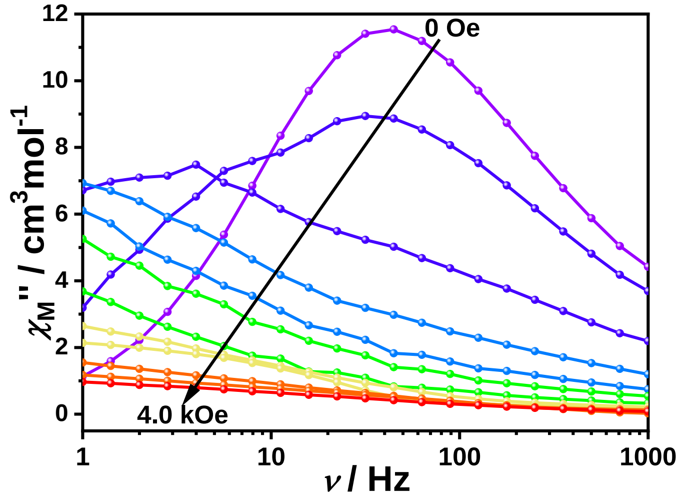

Figure S10 Isothermal field sweep measurement performed on polycrystalline sample of complex **1**.

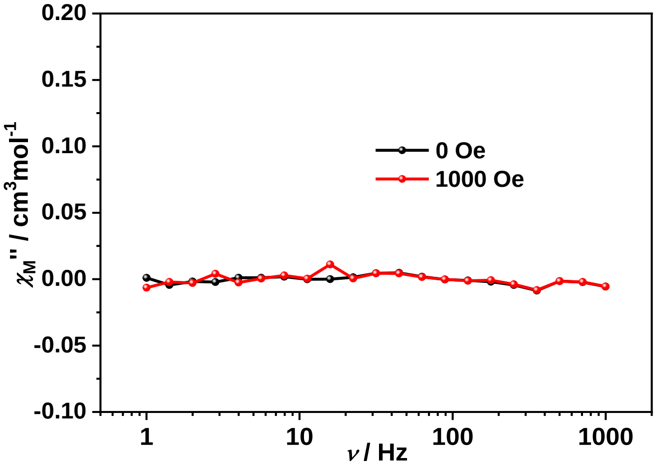

Figure S11 Isothermal field sweep measurement performed on polycrystalline sample of complex **1a**.

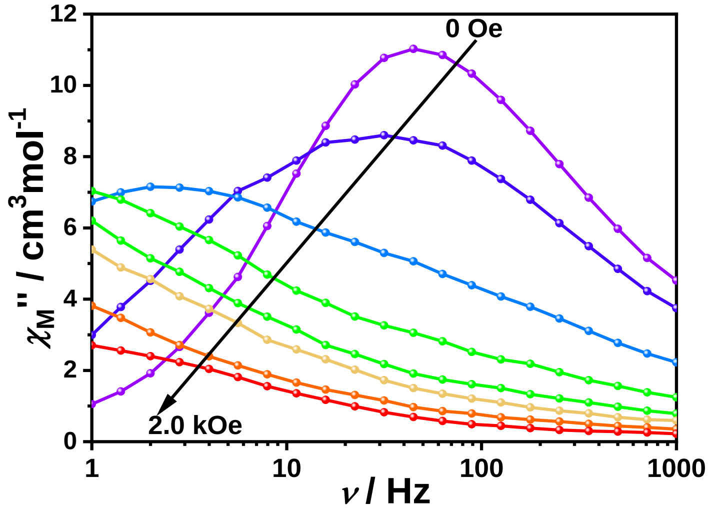

Figure S12 Isothermal field sweep measurement performed on polycrystalline sample of complex **1-back**.

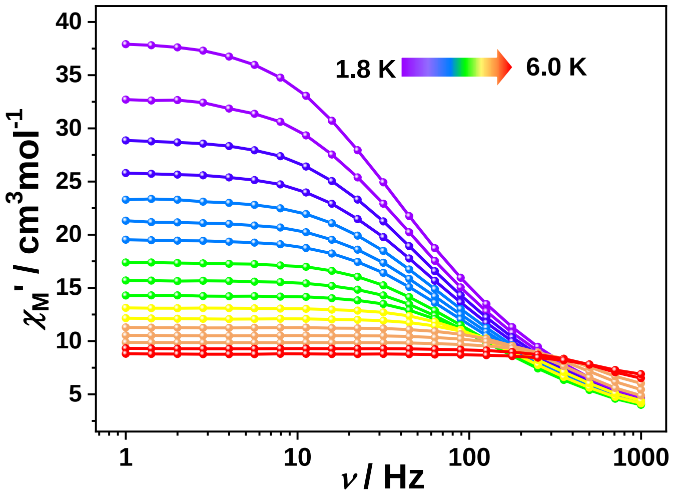

Figure S13 Frequency-dependent of in-put phase (χ_M') AC susceptibilities for **1** under zero field.

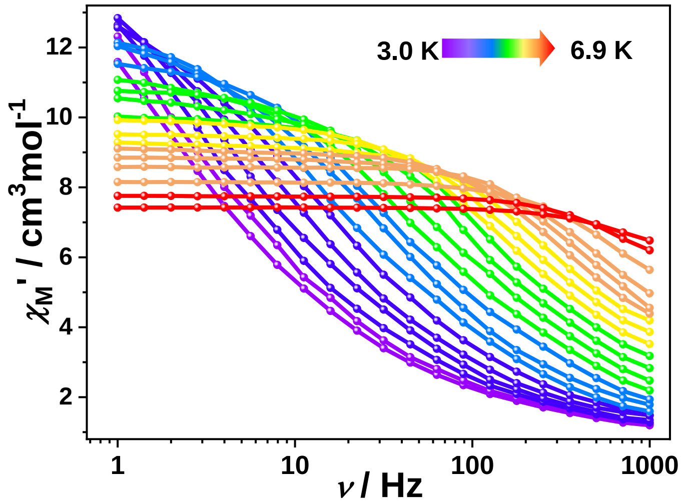

Figure S14 Frequency-dependent of in-put phase (χ_M') AC susceptibilities for **1** under 1.0 kOe field.

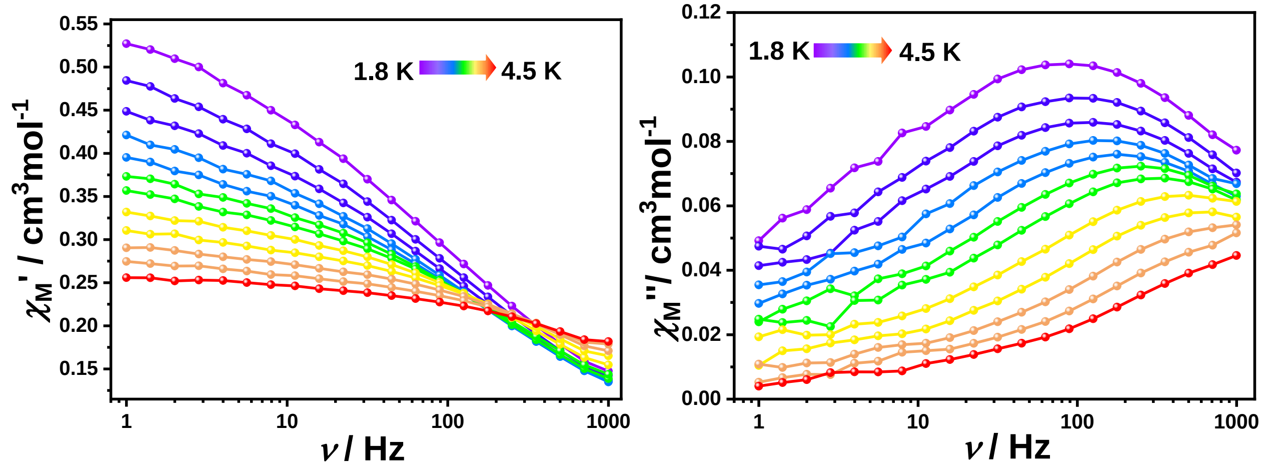

Figure S15 Frequency-dependent of χ_M' (left) and χ_M'' (right) for complex **1@Y** under zero field.

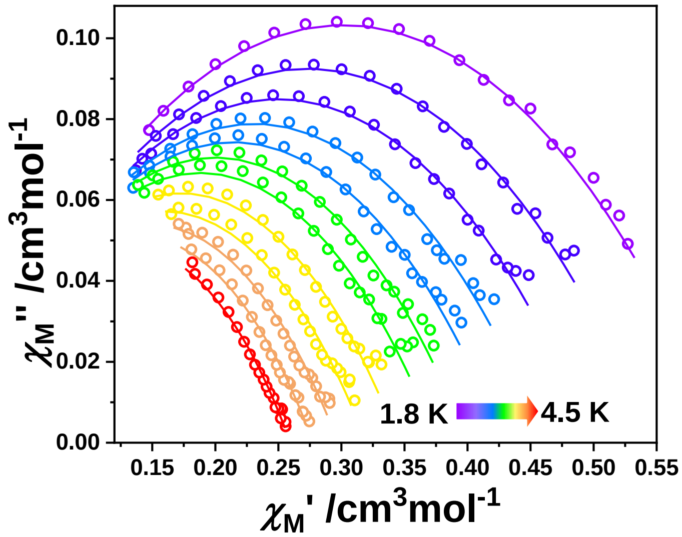

Figure S16 Cole−Cole curves for **1@Y** under zero field. Solid lines represent the best fit with Debye model.

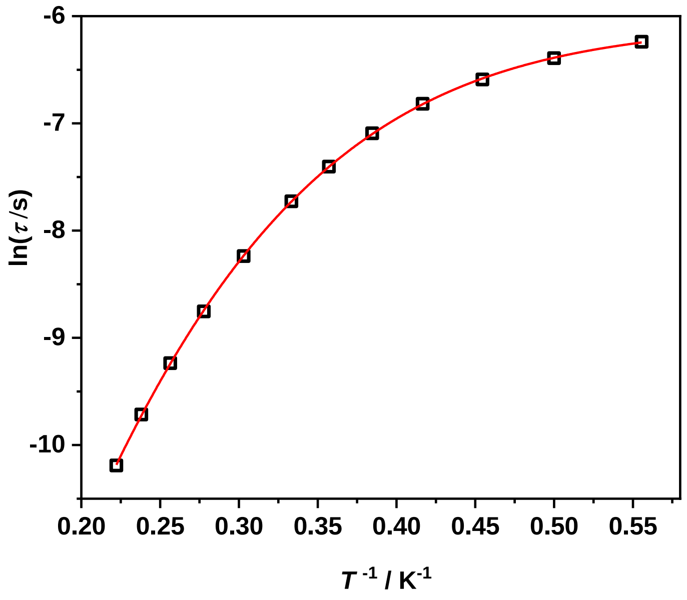

Figure S17 Plot of ln(τ / s) versus T^{-1} for complex **1@Y** under zero field, where the red solid line represents the fitted results using Equation (1).

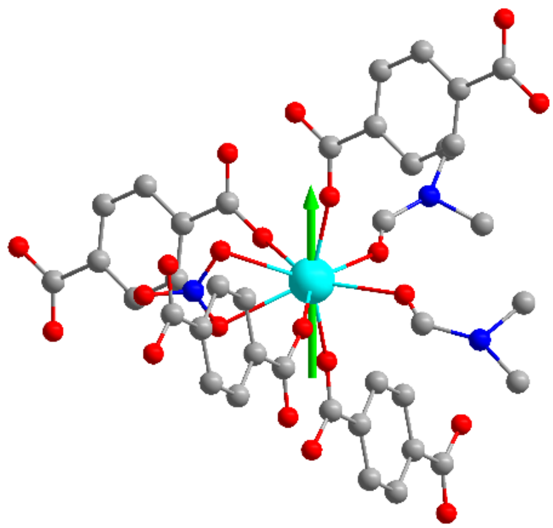

Figure S18 The ab initio calculated orientation of the local main magnetic axe of the ground KDs on individual Dy(III) ion for complex **1**. Colour code: Dy, light-blue; O, red; N, blue; C, gray. Hydrogen atoms are omitted for clarity.

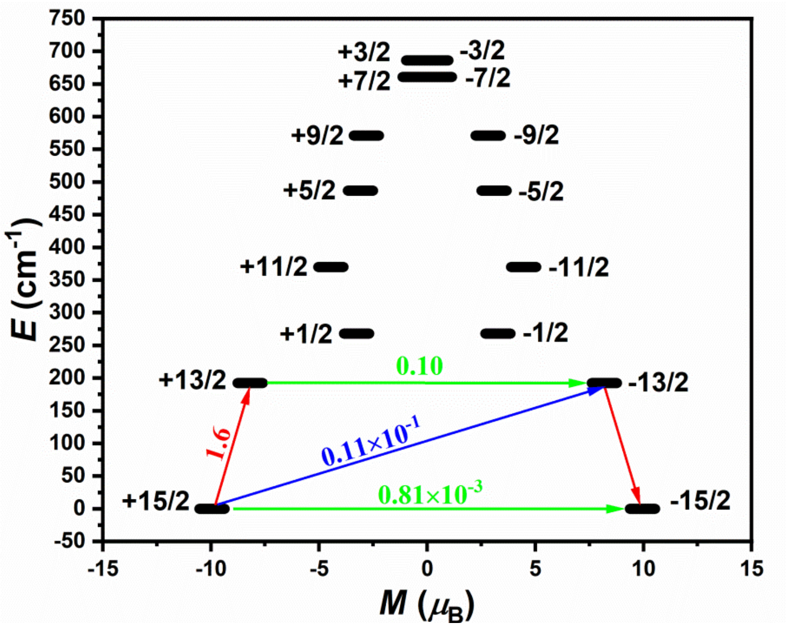

Figure S19 The Magnetization blocking barriers in complex **1**. The blue lines represent the KDs as a function of their magnetic moments along the magnetic axis. The green lines correspond to diagonal QTM, and the blue line represents the Orbach relaxation process. The red arrows represent the most possible path for magnetic relaxation. The numbers at each arrow stand for the mean absolute values of the transversal magnetic moments.

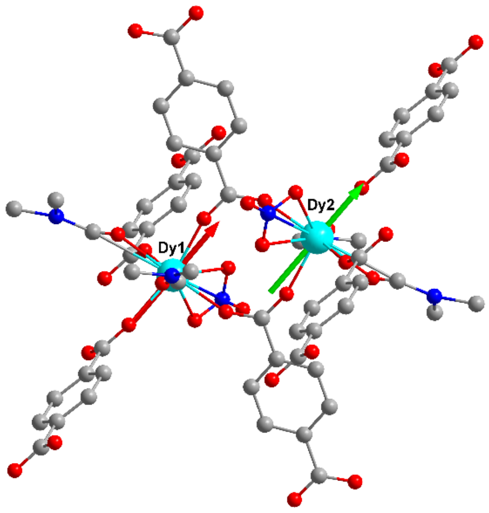

Figure S20 The main magnetic axes of two neighboring Dy (III) sites are parallel to each other. Colour code: Dy, light-blue; O, red; N, blue; C, gray. Hydrogen atoms are omitted for clarity.

The magnetic coupling between neighbouring Dy(III) ions for complex **1** was calculated with equation 1.

$$\boldsymbol{J_{dip}} = -\frac{\boldsymbol{\mu_B^2 g_{1Z} g_{2Z}}}{\boldsymbol{r^3}}(\boldsymbol{\cos\theta - 3\cos\varphi_1\cos\varphi_2}) \qquad (\boldsymbol{q})$$

where: θ − the angle between the main anisotropy axes on Dy1 site and Dy2 site; φ_1 − the angle between the main magnetic axis on Dy1 site with the unit vector connecting Dy1 site and Dy2 site (n_{12}); φ_2 − the angle between the main magnetic axis on Dy1 site with the unit vector connecting Dy1 site and Dy2 site (n_{12}); g_{1Z} − the Z component of the ground g tensor on Dy1 site; g_{2Z} − the Z component of the ground g tensor on Dy2 site; μ_B^2 − constant, = 0.4329701512063995 in units of cm^{-1}/Tesla; r − the distance between the Dy1 and Dy2, in Angstrom.

According the structure and calculation, we can obtain the values of the parameters, such as $\theta = 0$, $\varphi_1 = \varphi_2 = 34.672$ °, $g_{1Z} = g_{2Z} = 19.893$, $r = 5.425$ Å, and then obtain the value of $J_{dip} = 0.48\ cm^{-1}$.

Reference

1. Zhang, X.; Vieru, V.; Feng, X.; Liu, J.-L.; Zhang, Z.; Na, B.; Shi, W.; Wang, B.-W.; Powell, A. K.; Chibotaru, L. F.; Gao, S.; Cheng, P.; Long, J. R., Influence of Guest Exchange on the Magnetization Dynamics of Dilanthanide Single-Molecule-Magnet Nodes within a Metal–Organic Framework. *Angew.Chem. Int.Ed.* **2015,** *54* (34), 9861-9865.
2. Zhou, Q.; Yang, F.; Xin, B.; Zeng, G.; Zhou, X.; Liu, K.; Ma, D.; Li, G.; Shi, Z.; Feng, S., Reversible switching of slow magnetic relaxation in a classic lanthanide metal-organic framework system. *Chem. Commun.* **2013,** *49* (74), 8244-6.
3. Zhang, S.; Ke, H.; Liu, X.; Wei, Q.; Xie, G.; Chen, S., A nine-coordinated dysprosium(III) compound with an oxalate-bridged dysprosium(III) layer exhibiting two slow magnetic relaxation processes. *Chem. Commun.* **2015,** *51* (82), 15188-91.
4. Liu, C. M.; Zhang, D. Q.; Zhu, D. B., A 3D MOF constructed from dysprosium(III) oxalate and capping ligands: ferromagnetic coupling and field-induced two-step magnetic relaxation. *Chem. Commun.* **2016,** *52* (26), 4804-7.
5. Wang, M.; Meng, X.; Song, F.; He, Y.; Shi, W.; Gao, H.; Tang, J. K.; Peng, C., Reversible structural transformation induced switchable single-molecule magnet behavior in lanthanide metal–organic frameworks. *Chem. Commun.* **2018,** *54* (72), 10183-10186.
6. Baldovi, J. J.; Coronado, E.; Gaita-Arino, A.; Gamer, C.; Gimenez-Marques, M.; Minguez Espallargas, G., A SIM-MOF: three-dimensional organisation of single-ion magnets with anion-exchange capabilities. *Chem. Eur. J.* **2014,** *20* (34), 10695-702.
7. Na, B.; Zhang, X. J.; Shi, W.; Zhang, Y. Q.; Wang, B. W.; Gao, C.; Gao, S.; Cheng, P., Six-coordinate lanthanide complexes: slow relaxation of magnetization in the dysprosium(III) complex. *Chem. Eur. J.* **2014,** *20* (48), 15975-80.
8. Huang, G.; Fernandez-Garcia, G.; Badiane, I.; Camarra, M.; Freslon, S.; Guillou, O.; Daiguebonne, C.; Totti, F.; Cador, O.; Guizouarn, T.; Le Guennic, B.; Bernot, K., Magnetic Slow Relaxation in a Metal-Organic Framework Made of Chains of Ferromagnetically Coupled Single-Molecule Magnets. *Chem. Eur.J.* **2018,** *24* (27), 6983-6991.
9. Yin, D. D.; Chen, Q.; Meng, Y. S.; Sun, H. L.; Zhang, Y. Q.; Gao, S., Slow magnetic relaxation in a novel carboxylate/oxalate/hydroxyl bridged dysprosium layer. *Chem Sci* **2015,** *6* (5), 3095-3101.
10. Li, Z.-Y.; Zhai, B.; Li, S.-Z.; Cao, G.-X.; Zhang, F.-Q.; Zhang, X.-F.; Zhang, F.-L.; Zhang, C., Two Series of Lanthanide Coordination Polymers with 2-Methylenesuccinate: Magnetic Refrigerant, Slow Magnetic Relaxation, and Luminescence Properties. *Cryst. Growth Des.* **2016,** *16* (8), 4574-4581.
11. Li, Z.-Y.; Cao, Y.-Q.; Li, J.-Y.; Zhang, X.-F.; Zhai, B.; Zhang, C. C.; Zhang, F.-L.; Cao, G.-X.,

Three Types of Lanthanide Coordination Polymers with Methylmalonate and Isonicotinate as Coligands: Structures, Luminescence, and Magnetic Properties. *Cryst. Growth Des.* **2017,** *17* (12), 6752-6761.

12. Oyarzabal, I.; Fernández, B.; Cepeda, J.; Gómez-Ruiz, S.; Calahorro, A. J.; Seco, J. M.; Rodríguez-Diéguez, A., Slow relaxation of magnetization in 3D-MOFs based on dysprosium dinuclear entities bridged by dicarboxylic linkers. *CrystEngComm* **2016,** *18* (17), 3055-3063.
13. Liu, Q.-Y.; Li, Y.-L.; Wang, Y.-L.; Liu, C.-M.; Ding, L.-W.; Liu, Y., Ionothermal synthesis of a 3D dysprosium–1,4-benzenedicarboxylate framework based on the 1D rod-shaped dysprosium–carboxylate building blocks exhibiting slow magnetization relaxation. *CrystEngComm* **2014,** *16* (3), 486-491.
14. Ma, X.; Xu, N.; Gao, C.; Li, L.; Wang, B.; Shi, W.; Cheng, P., Lanthanide hydroxide ribbons assembled in a 2D network: slow relaxation of the magnetization in the dysprosium(III) complex. *Dalton Trans.* **2015,** *44* (12), 5276-9.
15. Zhao, J.; Zhu, G. H.; Xie, L. Q.; Wu, Y. S.; Wu, H. L.; Zhou, A. J.; Wu, Z. Y.; Wang, J.; Chen, Y. C.; Tong, M. L., Magnetic and luminescent properties of lanthanide coordination polymers with asymmetric biphenyl-3,2',5'-tricarboxylate. *Dalton Trans.* **2015,** *44* (32), 14424-35.
16. Chen, Z.; Fang, M.; Kang, X. M.; Hou, Y. L.; Zhao, B., Assembly of single molecular magnets from dinuclear to 2D Dy-compounds with significant change of relaxation energy barriers. *Dalton Trans.* **2016,** *45* (1), 85-8.
17. Castells-Gil, J.; Baldoví, J. J.; Martí-Gastaldo, C.; Mínguez Espallargas, G., Implementation of slow magnetic relaxation in a SIM-MOF through a structural rearrangement. *Dalton Trans.* **2018,** *47* (41), 14734-14740.
18. Savard, D.; Lin, P. H.; Burchell, T. J.; Korobkov, I.; Wernsdorfer, W.; Clerac, R.; Murugesu, M., Two-dimensional networks of lanthanide cubane-shaped dumbbells. *Inorg. Chem.* **2009,** *48* (24), 11748-54.
19. Chen, M.; Sanudo, E. C.; Jimenez, E.; Fang, S. M.; Liu, C. S.; Du, M., Lanthanide-organic coordination frameworks showing new 5-connected network topology and 3D ordered array of single-molecular magnet behavior in the Dy case. *Inorg. Chem.* **2014,** *53* (13), 6708-14.
20. Yi, X.; Calvez, G.; Daiguebonne, C.; Guillou, O.; Bernot, K., Rational Organization of Lanthanide-Based SMM Dimers into Three-Dimensional Networks. *Inorg. Chem.* **2015,** *54* (11), 5213-9.
21. Hu, F. L.; Jiang, F. L.; Zheng, J.; Wu, M. Y.; Pang, J. D.; Hong, M. C., Magnetic Properties of 3D Heptanuclear Lanthanide Frameworks Supported by Mixed Ligands. *Inorg. Chem.* **2015,** *54* (13), 6081-3.
22. Gupta, S. K.; Bhat, G. A.; Murugavel, R., Lanthanide Organophosphate Spiro Polymers: Synthesis, Structure, and Magnetocaloric Effect in the Gadolinium Polymer. *Inorg. Chem.* **2017,** *56* (15), 9071-9083.
23. Li, Y.; Zhao, P.; Zhang, S.; Li, R.; Zhang, Y. Q.; Yang, E. C.; Zhao, X. J., A Rare Water and Hydroxyl-Extended One-Dimensional Dysprosium(III) Chain and Its Magnetic Dilution Effect. *Inorg. Chem. Front.* **2017,** *56* (16), 9594-9601.
24. Ma, F.; Chen, Q.; Xiong, J.; Sun, H. L.; Zhang, Y. Q.; Gao, S., Modulating Slow Magnetic Relaxation of Dysprosium Compounds through the Position of Coordinating Nitrate Group. *Inorg. Chem.* **2017,** *56* (21), 13430-13436.
25. Das, C.; Upadhyay, A.; Ansari, K. U.; Ogiwara, N.; Kitao, T.; Horike, S.; Shanmugam, M., Lanthanide-Based Porous Coordination Polymers: Syntheses, Slow Relaxation of Magnetization, and Magnetocaloric Effect. *Inorg. Chem.* **2018,** *57* (11), 6584-6598.
26. Liu, C.-M.; Zhang, D.-Q.; Zhao, Y.-S.; Hao, X.; Zhu, D.-B., Two-step warming solvothermal

syntheses, luminescence and slow magnetic relaxation of isostructural dense LnMOFs based on nanoscale 3-connected linkers. *Inorg. Chem. Front.* **2016,** *3* (8), 1076-1081.
27. Zhang, X.; Xu, N.; Shi, W.; Wang, B.-W.; Cheng, P., The influence of an external magnetic field and magnetic-site dilution on the magnetization dynamics of a coordination network based on ferromagnetic coupled dinuclear dysprosium(iii) units. *Inorg. Chem. Front.* **2018,** *5* (2), 432-437.
28. Ji, X.-Q.; Ma, F.; Xiong, J.; Yang, J.; Sun, H.-L.; Zhang, Y.-Q.; Gao, S., A rare chloride-bridged dysprosium chain with slow magnetic relaxation: a thermally activated mechanism via a second-excited state promoted by magnetic interactions. *Inorg. Chem. Front.* **2019,** *6* (3), 786-790.
29. Liu, C.-M.; Zhang, D.-Q.; Zhu, D.-B., Slow magnetic relaxation of a three-dimensional metal–organic framework featuring a unique dysprosium(iii) oxalate layer. *RSC Adv.* **2015,** *5* (78), 63186-63192.
30. Liu, C.-M.; Zhang, D.-Q.; Hao, X.; Zhu, D.-B., Luminescence and slow magnetic relaxation of isostructural 2D lanthanide metal-organic frameworks derived from both nicotinate N-oxide and glutarate. *Rsc Adv.* **2015,** *5* (113), 92980-92987.
31. Liu, C.-M.; Zhang, D.-Q.; Zhu, D.-B., A 2D → 2D polyrotaxane lanthanide–organic framework showing field-induced single-molecule magnet behaviour. *RSC Adv.* **2014,** *4* (68), 36053-36056.
32. Liu, C.-M.; Xiong, J.; Zhang, D.-Q.; Wang, B.-W.; Zhu, D.-B., Multiple thermal magnetic relaxation in a two-dimensional ferromagnetic dysprosium(iii) metal–organic framework. *RSC Adv.* **2015,** *5* (127), 104854-104861.
