# Supplementary material for: Reversible Switching of Single-Molecule Magnetic Behaviour by Desorption/Adsorption of Solvent Ligand in a New Dy(III)-Based Metal Organic Framework
Source: Front Chem. 2021 Aug 5;9:714851. doi: 10.3389/fchem.2021.714851 (PMC8374150; doi:10.3389/fchem.2021.714851)

checkCIF/PLATON report

Structure factors have been supplied for datablock(s) 1_a_pl

THIS REPORT IS FOR GUIDANCE ONLY. IF USED AS PART OF A REVIEW PROCEDURE FOR PUBLICATION, IT SHOULD NOT REPLACE THE EXPERTISE OF AN EXPERIENCED CRYSTALLOGRAPHIC REFEREE.

No syntax errors found. CIF dictionary Interpreting this report

Datablock: 1_a_pl

```
Bond precision:  C-C = 0.0082 A          Wavelength=0.71073

Cell:             a=17.13(4)     b=10.80(3)        c=10.69(4)
                  alpha=90       beta=95.80(4)     gamma=90
Temperature:      296 K

                Calculated                 Reported
Volume          1968(10)                   1968(10)
Space group     C 2/c                      C 2/c
Hall group      -C 2yc                     -C 2yc
Moiety formula  C14 H18 Dy N3 O9           ?
Sum formula     C14 H18 Dy N3 O9           C14 H18 Dy N3 O9
Mr              534.81                     534.81
Dx,g cm-3       1.805                      1.805
Z               4                          4
Mu (mm-1)       3.846                      3.847
F000            1044.0                     1044.0
F000'           1043.78
h,k,lmax        20,12,12                   20,12,12
Nref            1734                       1722
Tmin,Tmax       0.529,0.655                0.147,0.261
Tmin'           0.515

Correction method= # Reported T Limits: Tmin=0.147 Tmax=0.261
AbsCorr = MULTI-SCAN

Data completeness= 0.993          Theta(max)= 25.009

R(reflections)= 0.0279( 1654)     wR2(reflections)= 0.0692( 1722)

S = 1.063                 Npar= 199
```

The following ALERTS were generated. Each ALERT has the format
test-name_ALERT_alert-type_alert-level.
Click on the hyperlinks for more details of the test.

Alert level C

```
ABSTY02_ALERT_1_C  An _exptl_absorpt_correction_type has been given without
            a literature citation. This should be contained in the
            _exptl_absorpt_process_details field.
            Absorption correction given as multi-scan
SHFSU01_ALERT_2_C  The absolute value of parameter shift to su ratio > 0.05
            Absolute value of the parameter shift to su ratio given   0.059
            Additional refinement cycles may be required.
PLAT080_ALERT_2_C Maximum Shift/Error ............................      0.06 Why ?
PLAT088_ALERT_3_C Poor Data / Parameter Ratio ....................      8.65 Note
PLAT148_ALERT_3_C s.u. on the      a    - Axis is (Too) Large ....     0.040 Ang.
PLAT148_ALERT_3_C s.u. on the      b    - Axis is (Too) Large ....    0.0300 Ang.
PLAT148_ALERT_3_C s.u. on the      c    - Axis is (Too) Large ....     0.040 Ang.
PLAT213_ALERT_2_C Atom O1              has ADP max/min Ratio .....       3.1 prolat
PLAT220_ALERT_2_C Non-Solvent  Resd 1  O   Ueq(max)/Ueq(min) Range       4.0 Ratio
PLAT241_ALERT_2_C High   'MainMol' Ueq as Compared to Neighbors of        O2 Check
PLAT242_ALERT_2_C Low    'MainMol' Ueq as Compared to Neighbors of       Dy1 Check
PLAT242_ALERT_2_C Low    'MainMol' Ueq as Compared to Neighbors of        N1 Check
PLAT342_ALERT_3_C Low Bond Precision on  C-C Bonds ...............   0.00825 Ang.
PLAT911_ALERT_3_C Missing FCF Refl Between Thmin & STh/L=    0.595        12 Report
PLAT977_ALERT_2_C Check Negative Difference Density on H2C             -0.36 eA-3
```

Alert level G

```
PLAT002_ALERT_2_G Number of Distance or Angle Restraints on AtSite         9 Note
PLAT003_ALERT_2_G Number of Uiso or Uij Restrained non-H Atoms ...        15 Report
PLAT004_ALERT_5_G Polymeric Structure Found with Maximum Dimension         3 Info
PLAT083_ALERT_2_G SHELXL Second Parameter in WGHT  Unusually Large      5.15 Why ?
PLAT171_ALERT_4_G The CIF-Embedded .res File Contains EADP Records         1 Report
PLAT172_ALERT_4_G The CIF-Embedded .res File Contains DFIX Records         2 Report
PLAT174_ALERT_4_G The CIF-Embedded .res File Contains FLAT Records         3 Report
PLAT178_ALERT_4_G The CIF-Embedded .res File Contains SIMU Records         1 Report
PLAT186_ALERT_4_G The CIF-Embedded .res File Contains ISOR Records         1 Report
PLAT301_ALERT_3_G Main Residue  Disorder ..............(Resd  1  )       36% Note
PLAT380_ALERT_4_G Incorrectly? Oriented X(sp2)-Methyl Moiety .....       C2A Check
PLAT380_ALERT_4_G Incorrectly? Oriented X(sp2)-Methyl Moiety .....       C3A Check
PLAT432_ALERT_2_G Short Inter X...Y Contact  O1       ..C3              2.94 Ang.
                                               x,-1+y,z  =      1_545 Check
PLAT432_ALERT_2_G Short Inter X...Y Contact  O1       ..C3              2.94 Ang.
                                         1-x,-1+y,1/2-z  =      2_645 Check
PLAT720_ALERT_4_G Number of Unusual/Non-Standard Labels ..........        14 Note
PLAT811_ALERT_5_G No ADDSYM Analysis: Too Many Excluded Atoms ....         ! Info
PLAT860_ALERT_3_G Number of Least-Squares Restraints .............       614 Note
PLAT883_ALERT_1_G No Info/Value for _atom_sites_solution_primary .    Please Do !
PLAT909_ALERT_3_G Percentage of I>2sig(I) Data at Theta(Max) Still       93% Note
PLAT933_ALERT_2_G Number of OMIT Records in Embedded .res File ...         6 Note
PLAT978_ALERT_2_G Number C-C Bonds with Positive Residual Density.         3 Info
```

---

 0 **ALERT level A** = Most likely a serious problem - resolve or explain
 0 **ALERT level B** = A potentially serious problem, consider carefully
15 **ALERT level C** = Check. Ensure it is not caused by an omission or oversight
21 **ALERT level G** = General information/check it is not something unexpected

 2 ALERT type 1 CIF construction/syntax error, inconsistent or missing data
15 ALERT type 2 Indicator that the structure model may be wrong or deficient
 9 ALERT type 3 Indicator that the structure quality may be low
 8 ALERT type 4 Improvement, methodology, query or suggestion
 2 ALERT type 5 Informative message, check

It is advisable to attempt to resolve as many as possible of the alerts in all categories. Often the minor alerts point to easily fixed oversights, errors and omissions in your CIF or refinement strategy, so attention to these fine details can be worthwhile. In order to resolve some of the more serious problems it may be necessary to carry out additional measurements or structure refinements. However, the purpose of your study may justify the reported deviations and the more serious of these should normally be commented upon in the discussion or experimental section of a paper or in the "special_details" fields of the CIF. checkCIF was carefully designed to identify outliers and unusual parameters, but every test has its limitations and alerts that are not important in a particular case may appear. Conversely, the absence of alerts does not guarantee there are no aspects of the results needing attention. It is up to the individual to critically assess their own results and, if necessary, seek expert advice.

Publication of your CIF in IUCr journals

A basic structural check has been run on your CIF. These basic checks will be run on all CIFs submitted for publication in IUCr journals (*Acta Crystallographica*, *Journal of Applied Crystallography*, *Journal of Synchrotron Radiation*); however, if you intend to submit to *Acta Crystallographica Section C* or *E* or *IUCrData*, you should make sure that full publication checks are run on the final version of your CIF prior to submission.

Publication of your CIF in other journals

Please refer to the *Notes for Authors* of the relevant journal for any special instructions relating to CIF submission.

PLATON version of 03/05/2019; check.def file version of 29/04/2019

Datablock 1_a_pl - ellipsoid plot

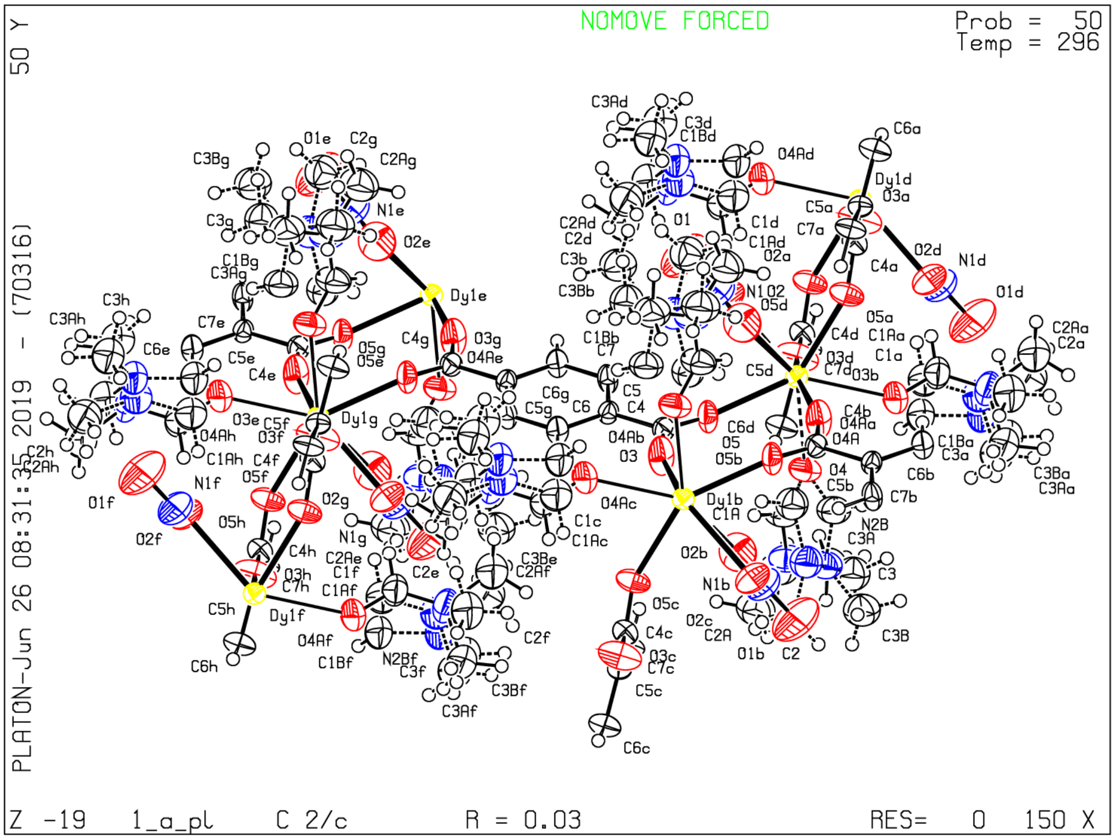

Supplement: Supplementary file 2 [file DataSheet1.ZIP › Crystal data and cifcheck/3-2 checkcif.pdf]
